# Bibliometric analysis of research trends and hotspots on robot-assisted thyroid surgery

**DOI:** 10.1097/MD.0000000000037588

**Published:** 2024-04-05

**Authors:** Xiao-Tong Shi, Jun Shen, Ya-Yu Sun

**Affiliations:** a Department of Head and Neck, Sir Run Run Shaw Hospital, Zhejiang University School of Medicine, Hangzhou, China; b Department of Surgical Oncology, Sir Run Run Shaw Hospital, Zhejiang University School of Medicine, Hangzhou, China; c Department of Ultrasound, Sir Run Run Shaw Hospital, Zhejiang University School of Medicine, Hangzhou, China.

**Keywords:** bibliometrics analysis, learning curve, robot-assisted thyroid surgery, transoral robotic thyroidectomy

## Abstract

**Background:**

Thyroid surgery involves the partial or complete removal of the thyroid gland and is a frequently performed surgical procedure. The adoption of robots, equipped with flexible and stable operating systems, has garnered acceptance among numerous surgeons for their capability to enable precise anatomical dissection in thyroid surgery. To gain a comprehensive insight into the present research landscape of robot-assisted thyroid surgery, this paper endeavored to conduct a thorough analysis of the field through bibliometric analysis.

**Methods:**

Relevant literature pertaining to robot-assisted thyroid surgery was retrieved from the Web of Science Core Collection (WOSCC) database, spanning from the inception of WOSCC to October 17, 2022. Visual analyses of publication quantity, distribution across countries/regions, institutions/organizations, authorship, journals, references, and keywords were conducted using Microsoft Excel, the bibliometrix package in R, Citescape, and VOSviewer software.

**Results:**

A total of 505 articles from 406 institutions in 36 countries/regions were included. South Korea emerged with highest number of publications. Notably, Professor CHUNG WY from Yonsei University in South Korea and the journal “*Surg Endosc*” stood out with the most publications. The current research landscape indicated significant interest in endoscopic thyroidectomy, surgical procedures, and the axillary approach. In addition, transoral robotic thyroidectomy (TROT), and learning curve (LC) were recognized as research frontiers, representing potential future hotspots in this field.

**Conclusion:**

This study marks the first bibliometric analysis of the literature on robot-assisted thyroid surgery. The results highlight endoscopic thyroidectomy, surgical procedures, and the axillary approach as current research hotspots, with TROT and LC identified as potential future research hotspots.

## 1. Introduction

Thyroid surgery is a surgical procedure involving the partial or complete removal of the thyroid gland. Open thyroidectomy, initially introduced by Professor Emil Theodor Kocher (1841–1917),^[1]^ has become a cornerstone in clinical practices for patients with thyroid tumors, moderate or severe hyperthyroidism not responsive to conservative treatment, or thyroid enlargement causing compressive symptoms.^[2,3]^ This surgical approach has demonstrated outstanding clinical outcomes, attributed to its clear surgical field and enhanced accessibility.^[4]^ In light of the growing number of young women seeking surgery for thyroid diseases with a preference for concealed neck scars,^[5,6]^ there has been a flourishing trend in minimally invasive and endoscopic techniques. Despite the maturation of endoscopic techniques over the years, they continue to pose certain limitations, including a restricted 2-dimensional visual field, compromised hand-eye coordination, and a challenging learning curve (LC).^[7]^ In addressing these limitations, the introduction of robot-assisted surgery with its unique advantages has emerged as a promising solution. The benefits of incorporating robotic technology encompass: implementation of a dual-channel stereoscopic endoscope system, ensuring a continuous and stable 3-dimensional magnified surgical field;^[8]^ integration of an automated tremor-filtering system that aids in the precise dissection and protection of critical structures, including the recurrent laryngeal nerves and parathyroid glands;^[9]^ utilization of multi-degree-of-freedom Endo-wrist robotic arms, facilitating more flexible and convenient surgical maneuvers;^[10]^ and deployment of multiple robotic arms that effectively expose anatomical layers, achieving a sophisticated “membrane dissection.”^[10]^ These distinctive advantages offered by robotic technology contribute to the advancement of thyroid surgery, promoting enhanced safety and scarless procedures, thereby fostering increased acceptance among patients.

Kang et al^[11]^ from South Korea were pioneers in extensively employing robot-assisted thyroidectomy via the axillary approach in clinical practice. Their assessment of pathological characteristics, postoperative hospital stay, and prognostic outcomes led to the conclusion that this approach is both safe and feasible. Following this groundbreaking work, numerous research teams have reported successful experiences with this technique. For instance, Lee et al,^[12]^ in clinical studies, demonstrated that robot-assisted thyroidectomy yielded superior cosmetic outcomes compared to open thyroidectomy, with reductions in postoperative neck discomfort and swallowing difficulties. In a comparative study between endoscopic thyroidectomy and robot-assisted thyroidectomy, Lang et al^[13]^ found that the latter required longer operating times but fewer surgical assistants. Since then, the literature on robot-assisted thyroid surgery has witnessed exponential growth, and surgical approaches have become more diverse, including axillary, breast, and transoral vestibular approaches. These developments advancements underscore increasing attention and recognition that robot-assisted thyroid surgery is garnering from clinical researchers.

Bibliometrics is a interdisciplinary field that draws on the theories and methodologies from metrology, statistics, and librarianship. It involves the quantitative analysis and categorization of literature within a specific field.^[14,15]^ This form of analysis allows for the visualization and examination of various aspects, including authors, keywords, institutions/organizations, countries/regions, journals, and cited references. It provides valuable insights into the global development and trends of a particular field. Currently, widely utilized bibliometric software includes the bibliometrix package in R 4.2.1, VOSviewer 1.6.18 and CiteSpace 6.1.R3.^[16,17]^ Researchers can leverage bibliometric analysis to swiftly comprehend the current status and trends in a specific field, thereby guiding future research endeavors. In this study, Microsoft Excel, the bibliometrix package in R software, VOSviewer, and CiteSpace software will be employed to analyze the literature on robot-assisted thyroid surgery from the past 2 decades, offering valuable references for researchers and clinical practitioners engaged in the advancement of robot-assisted thyroid surgery.

## 2. Materials and methods

### 2.1. Literature sources and retrieval strategies

Web of Science, one of the largest and most comprehensive electronic scientific literature databases globally, includes over 12,000 influential journals. When compared to other databases like Scopus and PubMed, Web of Science is widely acknowledged as the most commonly used, comprehensive, and reliable platform for bibliometric analysis.^[18]^ The literature is sourced from the Web of Science Core Collection database (WOSCC), with the retrieval conducted on October 17, 2022. The retrieval strategy is outlined as follows: TS =((Procedure, Robotic Surgical*) OR (Procedures, Robotic Surgical*) OR (Robotic Surgical Procedure*) OR (Surgical Procedure, Robotic*) OR (Robot Surgery*) OR (Robot Surgeries*) OR (Surgery, Robot*) OR (Robot-Assisted Surgery*) OR (Robot-Assisted Surgery*) OR (Robot-Assisted Surgeries*) OR (Surgery, Robot-Assisted*) OR (Robot-Enhanced Procedures*) OR (Procedure, Robot-Enhanced*) OR (Robot-Enhanced Procedures*) OR (Robot-Enhanced Procedure*) OR (Surgical Procedures, Robotic*) OR (Robotic-Assisted Surgery*) OR (Robotic-Assisted Surgery*) OR (Robotic-Assisted Surgeries*) OR (Surgery, Robotic-Assisted*) OR (Robot-Enhanced Surgery*) OR (Robot-Enhanced Surgery*) OR (Robot-Enhanced Surgeries*) OR (Surgery, Robot-Enhanced*)) AND TS = ((Thyroid*) OR (Thyroids*) OR (Thyroid Glands*) OR (Gland, Thyroid*) OR (Glands, Thyroid*)).

### 2.2. Inclusion and exclusion criteria

Inclusion criteria: Original research articles and review papers; Language limited to English. Exclusion criteria: duplicate publications; non-original research papers (letters, conference abstracts, book chapters, editorial materials, corrections, news articles, and retracted articles); and papers unrelated to robot-assisted thyroid surgery.

### 2.3. Data collection

The literature search adhered to the retrieval strategy outlined in section “2.1,” and the screening process followed the inclusion and exclusion criteria specified in section “2.2.” Two researchers (referred to as XTS and JS) independently performed these operations. In the event of any discrepancies, a third author (referred to as YYS) was consulted for resolution. All data was ultimately exported in download_txt format and stored in the data folder.

### 2.4. Data analysis

In this study, Microsoft Excel 2016, the bibliometrix package in R 4.2.1, VOSviewer 1.6.18, and CiteSpace 6.1.R3 were used for visual analysis of the data. The following aspects were scrutinized: the annual publication count, distribution by countries/regions, affiliations of institutions/organizations, prolific authors, prominent journals, citation counts, global citation score (GCS), local citation score (LCS), H-index^[19]^ (assessing academic contributions and predicting future scientific achievements), G-index^[19]^ (addressing the limitation of H-index by accounting for authors with few publications but high citation frequencies), and M-index^[19]^ (addressing the limitation of H-index by considering insufficient time). Finally, the bibliometrix package in R, VOSviewer, and CiteSpace were utilized to visualize the distribution of countries/regions, institutions/organizations, authors, journals, cited references, and keywords.

The Bibliometrix package in the R software^[20]^ serves as an open-source tool specifically crafted for bibliometrics and quantitative science metrics research. This tool is capable of generating a range of performance indicators, encompassing the total number of publications, types of publication, publications originating from countries and institutions, the overall count of references and co-cited references, the total number of authors and co-cited authors, and the cumulative number of keywords. Moreover, it facilitates the identification of the top 10 countries/regions, institutions/organizations, and authors based on the highest publication count. It also highlights the most influential journals (top 6) and the most impactful papers (top 10). By utilizing the Bibliometrix package, researchers can conduct thorough analyses and visually explore publication trends across diverse dimensions, including years, countries/regions, institutions/organizations, authors, journals, and document types.

VOSViewer^[21]^ (Version 1.6.18) is a software tool designed for bibliometric mapping, text mining, and visualization in literature analysis. Through text mining, VOSviewer identifies scientific information embedded in the literature, including countries/regions, institutions/organizations, authors and co-cited authors, journals and co-cited journals, references, and keywords. It executes a visual mapping of similarities. In this research, the collected textual data will be imported into VOSviewer to construct a visual bibliometric network graph. Nodes represent entities such as countries/regions, institutions/organizations, journals, authors, and keywords. The size and color of nodes indicate the quantity and category of these entities, respectively. The thickness of lines between nodes reflects the degree of collaboration or co-citation, with nodes or lines of the same color signifying higher correlation.

CiteSpace (Version 6.2.R3), developed by Professor Chen,^[22]^ stands as another sophisticated software tool for bibliometric analysis and visualization. In this research, CiteSpace software is utilized to create dual-map overlays and conduct burst detection analyses for countries/regions, institutions/organizations, authors, and keywords. The specific parameter settings for CiteSpace are as follows: the time span ranges from January 2004 to October 2022, with each time slice representing 1 year. Node type is configured as references, selection criteria include g-index (k = 25), and pruning is performed using pathfinder, while other parameters remain at default values. The dual-map overlay illustrates the primary distribution areas of journals and co-cited journals, highlighting relationships between these areas (colors indicate different regions, and line thickness represents co-occurrence strength). Burst detection reveals periods of significant impact for countries/regions, institutions/organizations, authors, or keywords (bar segments denote years, with red bars indicating years of pronounced burst activity; the intensity reflects the strength of impact, and persistence signifies the most influential countries/regions, institutions/organizations, authors, or keywords based on the duration of bursts).

## 3. Results

### 3.1. Search results

**Figure 1.**
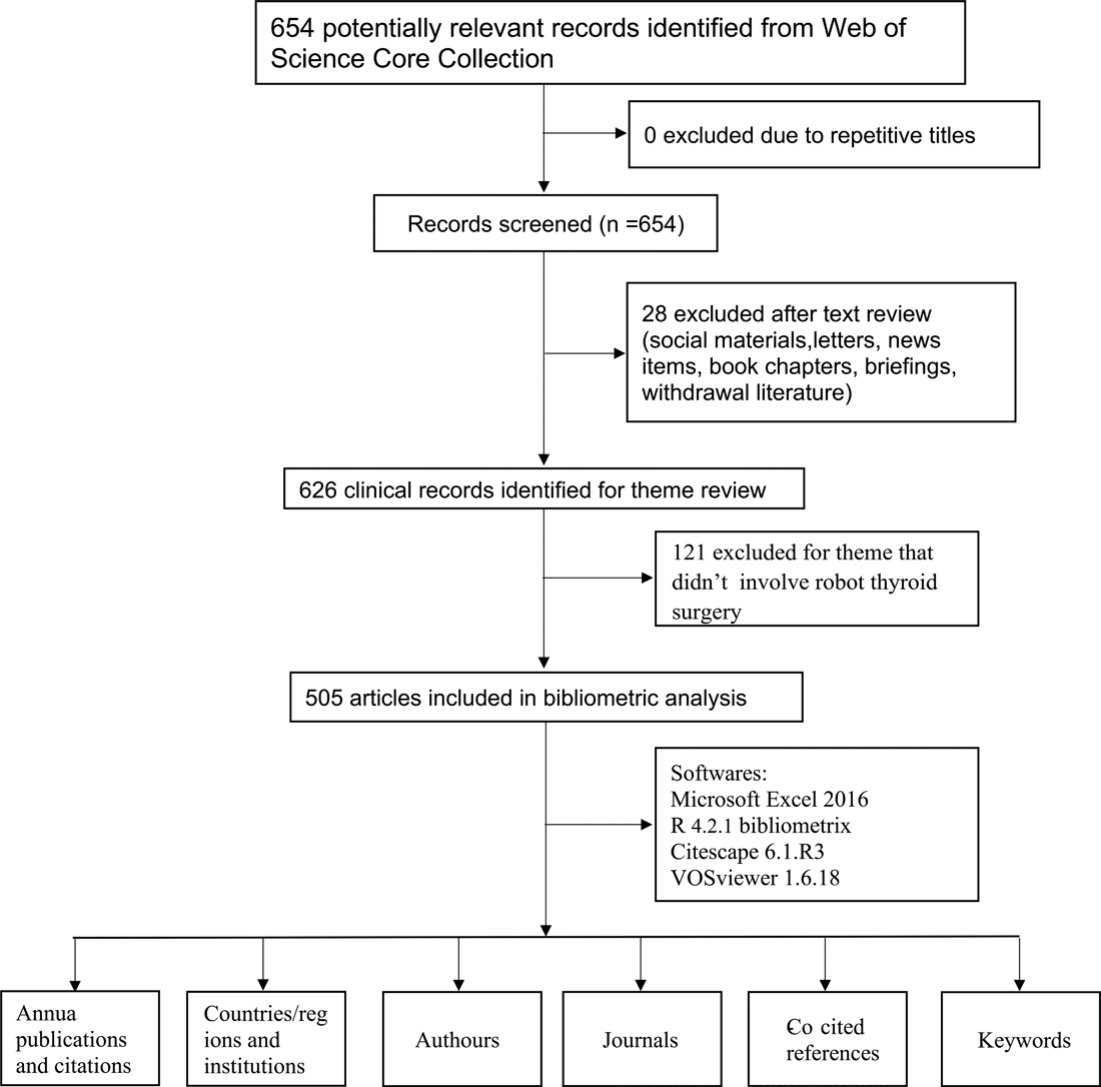
Database search flowchart.

An initial search in WOSCC resulted in 654 retrieved articles. Following the exclusion of 28 non-original articles and reviews, as well as 121 articles unrelated to robot-assisted thyroid surgery, a total of 505 articles were included, as depicted in Figure 1.

### 3.2. Annual publication volume and annual citation analysis

**Figure 2.**
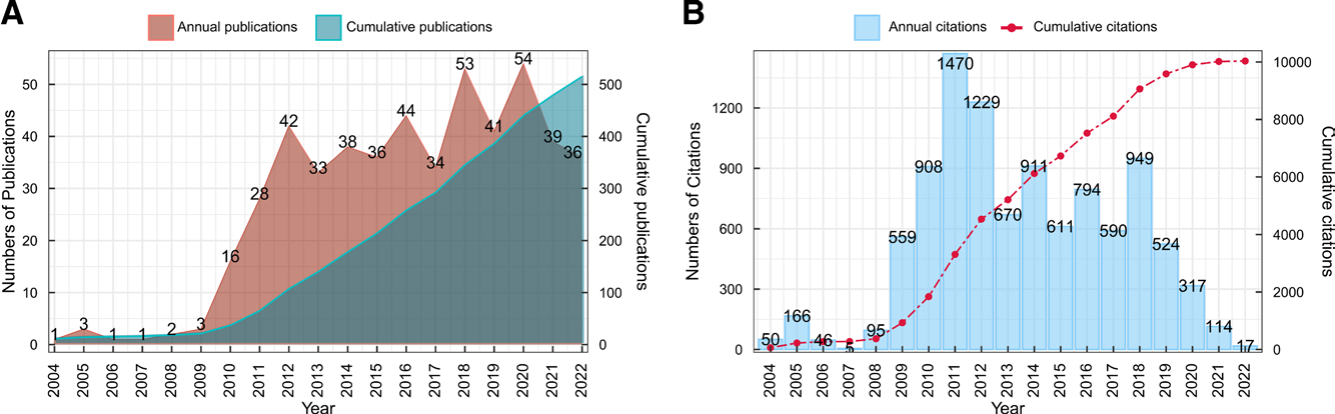
Annual publication trends. (A) Annual publication count and cumulative publication count; (B) Annual citation count and cumulative citation count.

The quantity of publications within a specific timeframe or region serves as an objective and quantitative reflection of the overall publishing trend during that period or within that area. The 505 included articles span the period from 2004 to 2022, which can be delineated into 2 stages, with 2009 serving as the pivotal turning point. The initial phase, spanning from 2004 to 2009, represents the early stage marked by a relatively modest number of publications (as low as 3 articles per year), indicating the nascent phase of research in robot-assisted thyroid surgery. The subsequent development phase, from 2010 to 2022, is characterized by a fluctuating and rapid increase in annual publication output. The zenith was attained in 2020 with 54 publications per year, followed by a marginal decrease (Fig. 2A). Figure 2B illustrates the annual citation count of robot-assisted thyroid surgery, confirming that 2009 indeed marked a turning point in the research field. In essence, these findings indicate the increasing attention of researchers and clinical practitioners toward the application of robot-assisted thyroid surgery in the field of thyroid surgery.

### 3.3. Distribution by countries/regions

**Figure 3.**
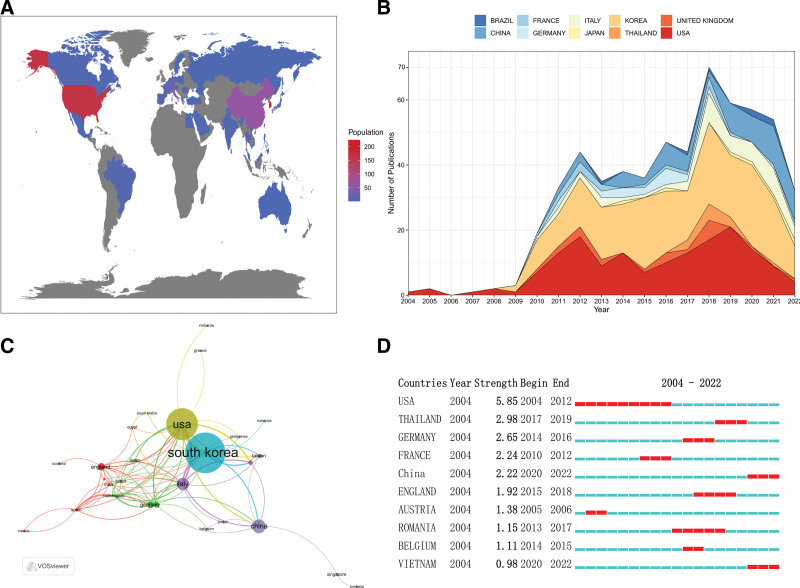
Contributions of different countries/regions to robotic thyroid surgery. (A) Publication output by different countries/regions worldwide; (B) Annual publication output of the top 10 countries/regions with the highest publication output; (C) Collaboration among countries/regions; (D) Emphasis analysis of the top 10 countries/regions with the highest citation intensity.

The corpus of 505 articles encompasses contributions from 36 countries/regions. Notably, South Korea emerged as the leader in publications (n = 224), trailed by the United States (n = 164), China (n = 64), Italy (n = 46), and the United Kingdom (n = 24). In terms of H-index, G-index, M-index, GCS, and LCS, both South Korea and the United States significantly surpassed other countries/regions. This delineates South Korea and the United States as the primary drivers of research activity in this field, steering global development of robotic thyroid surgery (Table 1, Fig. 3A). Figure 3B provided a visual representation of the annual publication trends among the top 10 countries contributing to research on robotic thyroid surgery from 2004 to 2022. The United States emerged as the earliest pioneer in this research, leading South Korea by a margin of 5 years, reaffirming its technological leadership on the global stage. Figure 3C delves into the collaboration dynamics among the 36 countries/regions. The size of the nodes corresponds to the publication output of each country/region, while the connections between nodes indicate the strength of collaboration. Notably, South Korea boasted the highest publication output, trailed by the United States and China, with the United States exhibiting the most robust international collaboration. In a concluding emphasis analysis on countries/regions, it became evident that the United States commands the highest citation intensity, reflecting its significant influence in the research field (Fig. 3D).

**Table 1:** Top 10 countries with the most publications.

Rank	Country	Publications	H-index	G-index	M-index	GCS	LCS
1	Korea	224	41	63	2.93	4961	2528
2	USA	164	37	56	1.95	3308	1449
3	China	64	17	23	1.42	486	221
4	Italy	46	15	30	1.15	178	52
5	United Kingdom	24	12	20	0.92	339	105
6	Germany	20	9	12	0.75	105	23
7	France	16	8	14	0.62	76	23
8	Thailand	12	10	23	1.67	128	17
9	Brazil	7	4	7	0.40	28	12
10	Japan	7	2	5	0.22	22	1

GCS = global citation score, LCS = local citation score.

### 3.4. Distribution of institutions/organizations

**Figure 4.**
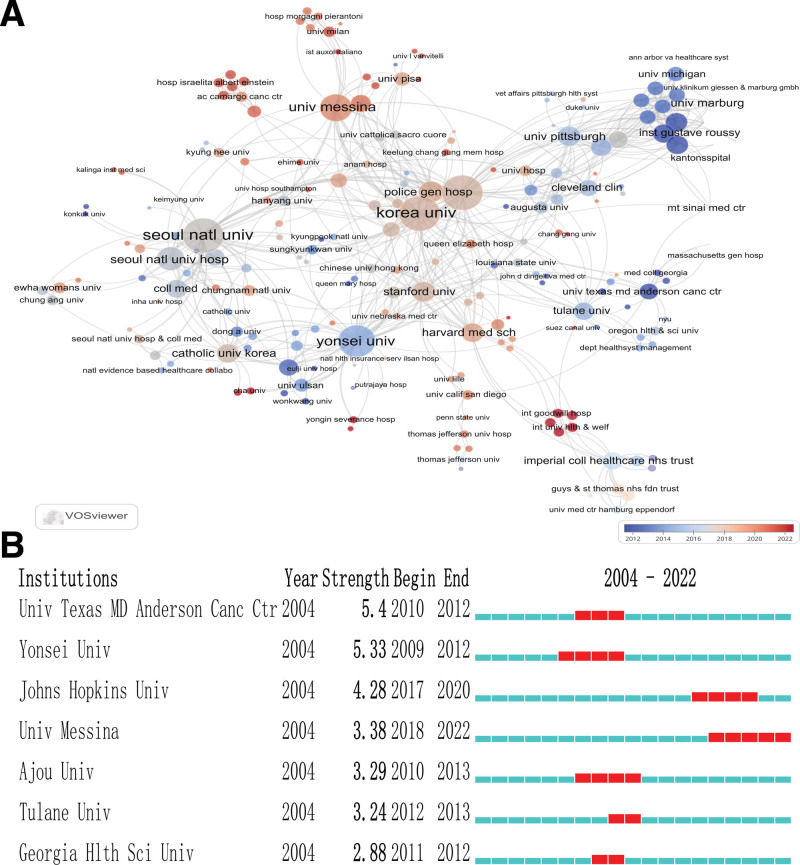
Different contributions of institutions/organizations to research on robot-assisted thyroid surgery. (A) Cooperation with global institutions/organizations; (B) Emphasis analysis of the top 10 institutions/organizations with the highest citation intensity.

A total of 406 institutions/organizations have contributed to research on robot-assisted thyroid surgery, with 49 of them publishing 5 or more papers. Table 2 summarized the top 10 institutions/organizations with the highest publication output, featuring 7 from South Korea, 2 from the United States, and 1 from the United Kingdom. Seoul National University in South Korea leaded with the highest number of publications at 108 papers, followed by Yonsei University from South Korea (84) and Hanyang University from South Korea (61). Yonsei University from South Korea also claimed the highest H-index, G-index, M-index, GCS, and LCS, signifying its substantial impact on robot-assisted thyroid surgery. Figure 4A illustrated the collaboration network among institutions/organizations, highlighting frequent collaboration between Seoul National University, Korea University, Johns Hopkins University in the United States, Yonsei University, and the University of Messina in Italy. Concluding the analysis, the prominence of institutions/organizations is examined, revealing that the University of Texas MD Anderson Cancer Center in the United States secured the top rank with a score of 5.4. It is followed by Yonsei University from South Korea (5.33), Johns Hopkins University in the United States (4.28), and the University of Messina in Italy (3.28), indicating their significant influence in the research field, as depicted in Figure 4B.

**Table 2:** The top 10 institutions/organizations with the most publications.

Rank	Affiliation	Country	Publications	H-index	G-index	M-index	GCS	LCS
1	Seoul Natl Univ	Korea	108	15	26	1.15	746	360
2	Yonsei Univ	Korea	84	28	50	2.00	2523	1267
3	Hanyang Univ	Korea	61	17	29	1.42	872	486
4	Tulane Univ	US	48	13	25	1.08	642	364
5	Korea Univ	Korea	39	18	30	1.38	917	306
6	Johns Hopkins Univ	US	32	18	27	1.20	735	242
7	Inje Univ	Korea	22	8	11	0.67	126	57
8	Catholic Univ Korea	Korea	20	7	15	0.64	235	127
9	Imperial Coll Healthcare Nhs Trust	United Kingdom	18	9	16	0.75	257	97
10	Seoul Natl Univ Hosp	Korea	16	9	16	0.75	243	120

GCS = global citation score, LCS = local citation score.

### 3.5. Authors and co-cited authors

**Figure 5.**
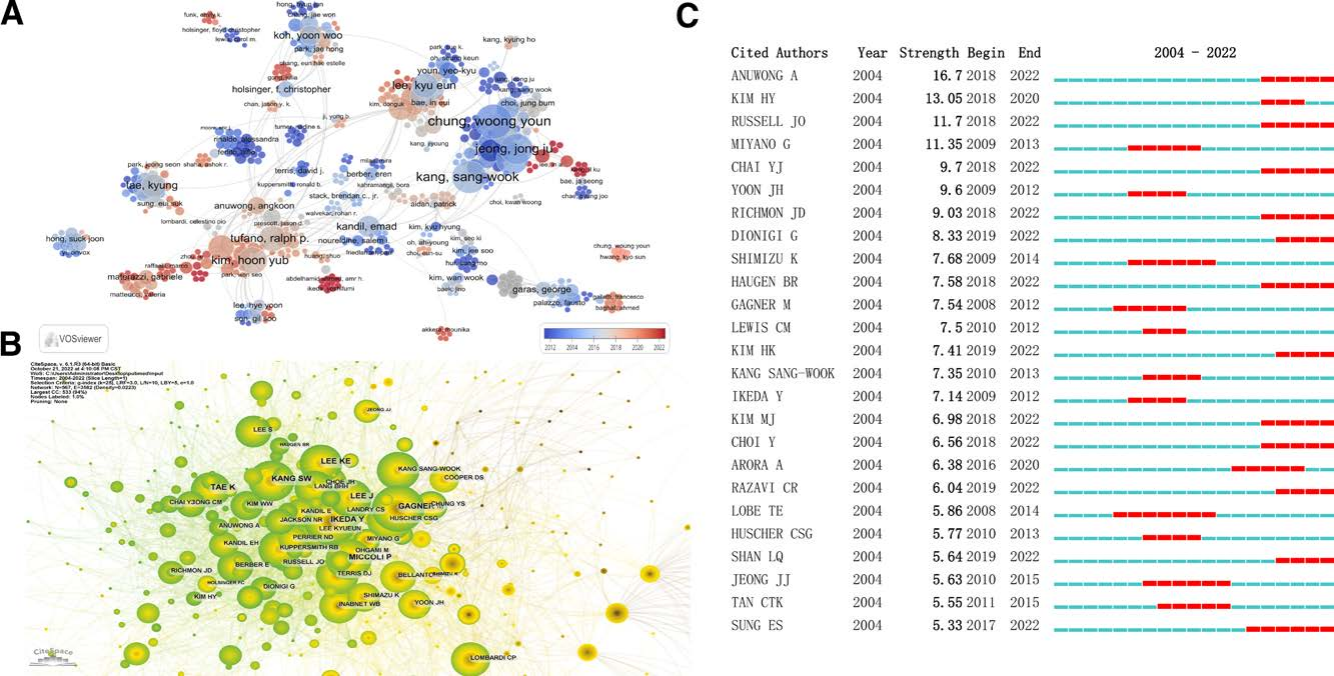
Contribution of authors to robot-assisted thyroid surgery. (A) Author collaboration network; (B) Co-cited author network; (C) Emphasis analysis of the top 25 authors with the highest citation frequency.

Since 2004, a total of 4183 authors have contributed to research on robot-assisted thyroid surgery. Among the top 10 most prolific authors, 8 were affiliated with Yonsei University, Hanyang University, and Korea University in South Korea, while 2 are from Emory University and Johns Hopkins Hospital in the United States. Professor CHUNG WY from Yonsei University in South Korea ranked first in terms of publication count, H-index, G-index, M-index, GCS, and LCS, highlighting their outstanding contributions to the field, as detailed in Table 3 and Figure 3. Figure 5A provided a visualization of the author collaboration network, with the highest-ranking teams being the CHUNG WY team from Yonsei University and the Kim, HY team from Korea University. The Kim, HY team closely collaborated with the TUFANO RP team from Johns Hopkins Hospital in the United States. Most authors appear as clustered nodes, signifying a relatively close collaboration among researchers and a wide reach of the teams. Co-cited authors, referring to authors simultaneously cited by at least one publication, indicate similarities in their research. The co-cited author network reveals that CHUNG WY had the highest co-citation count (227, centrality 0.02), followed by LEE J (169, centrality 0.04) and IKEDAY (137, centrality 0.05), suggesting that these 3 authors play a bridging role in the field of robot-assisted thyroid surgery, as depicted in Figure 5B. Furthermore, the emphasis analysis of co-cited authors highlighted that ANUWONG A had the highest citation intensity (16.7), followed by KIM HY (13.05), RUSSELL JO (11.7), and MIYANO G (11.35), indicating their significant influence in this research field, as demonstrated in Figure 5C.

**Table 3:** Top 10 authors with the most publications.

Rank	Author	Affiliation	Country	Publications	H-index	G-index	M-index	GCS	LCS
1	Chung WY	Yonsei Univ	Korea	47	27	47	1.93	2246	1214
2	Kang SW	Yonsei Univ	Korea	37	17	37	1.21	1454	786
3	Kim HY	Korea Univ	Korea	37	19	31	1.46	1002	366
4	Tae K	Hanyang Univ	Korea	34	17	29	1.42	872	486
5	Nam KH	Yonsei Univ	Korea	32	19	32	1.36	1594	864
6	Kandil E	Tulane Univ	US	31	12	19	1.00	408	217
7	Jeong JJ	Yonsei Univ	Korea	29	16	29	1.143	1364	756
8	Tufano RP	Johns Hopkins Univ Hosp	US	28	19	28	1.58	857	331
9	Ji YB	Hanyang Univ	Korea	27	16	27	1.33	792	458
10	Lee J	Yonsei Univ	Korea	24	16	24	1.23	965	538

GCS = global citation score, LCS = local citation score.

### 3.6. Journals and journal co-citation analysis

**Figure 6.**
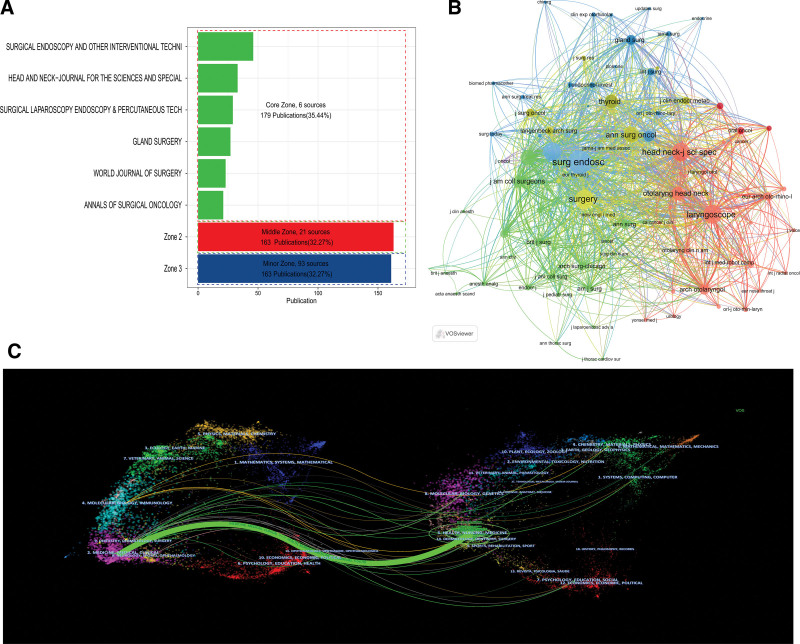
Contributions of journals to robot-assisted thyroid surgery research. (A) The relationship between different journals and the number of publications; (B) Co-citation network of journals; (C) Dual-map overlay of citing and cited journals.

A total of 505 articles were compiled from 120 journals. Among them, 179 articles (35.44%) originated from 6 core journals, while 163 articles (32.27%) were sourced from 21 other journals. The remaining 163 articles (32.27%) were obtained from 93 diverse journals, as illustrated in Figure 6A. Out of the 6 core journals, 4 hailed from the United States, while the remaining 2 were from Germany and China, respectively. The German journal “Surg Endosc” demonstrated significantly higher publication volume, LCS, and GCS compared to other journals, indicating its authoritative status in the field, as outlined in Table 4. Co-citation analysis of the journals unveiled that the top 4 co-cited journals were “*Surg Endosc*,” “*Surgery*,” “*Head Neck-J Sci Spec*,” and “*Laryngoscope*,” implying their substantial impact in the field, as depicted in Figure 6B. The overlay of dual maps in CiteSpace serves to reflect the research progress across different disciplines. As depicted in Figure 6C, the left side showcased citing journals, while the right side featured cited journals. The colored paths between them signified citation relationships, with green paths indicating that literature published in Health/Nursing/Medicine journals was frequently cited by Medicine/Medical/Clinical journals. Additionally, peripheral areas representing disciplines such as Physics/Materials/Chemistry, Ecology/Earth/Marine, Veterinary/Animal/Science, Mathematics/Systems/Mathematical, and Psychology/Education/Health also contribute to research on robot-assisted thyroid surgery, as illustrated in Figure 6C.

**Table 4:** Core journals for robotic thyroid surgery research.

Rank	Journal	Country	Publications	H-index	G-index	M-index	LCS	GCS	IF-2022	JCR
1	Surg Endosc	Germany	46	4	5	0.36	860	1618	3.453	Q2
2	Head Neck-J Sci Spec	US	33	13	21	1.08	421	833	3.821	Q1
3	Surg Laparo Endo Per	US	29	3	3	0.60	264	514	1.455	Q4
4	Gland Surgery	China	27	5	6	0.42	109	265	2.160	Q3
5	World J Surg	US	23	2	4	0.40	217	444	3.253	Q2
6	Ann Surg Oncol	US	21	9	15	1.29	365	678	4.339	Q1

GCS = global citation score, JCR = journal citation reports, LCS = local citation score.

### 3.7. Document analysis

**Figure 7.**
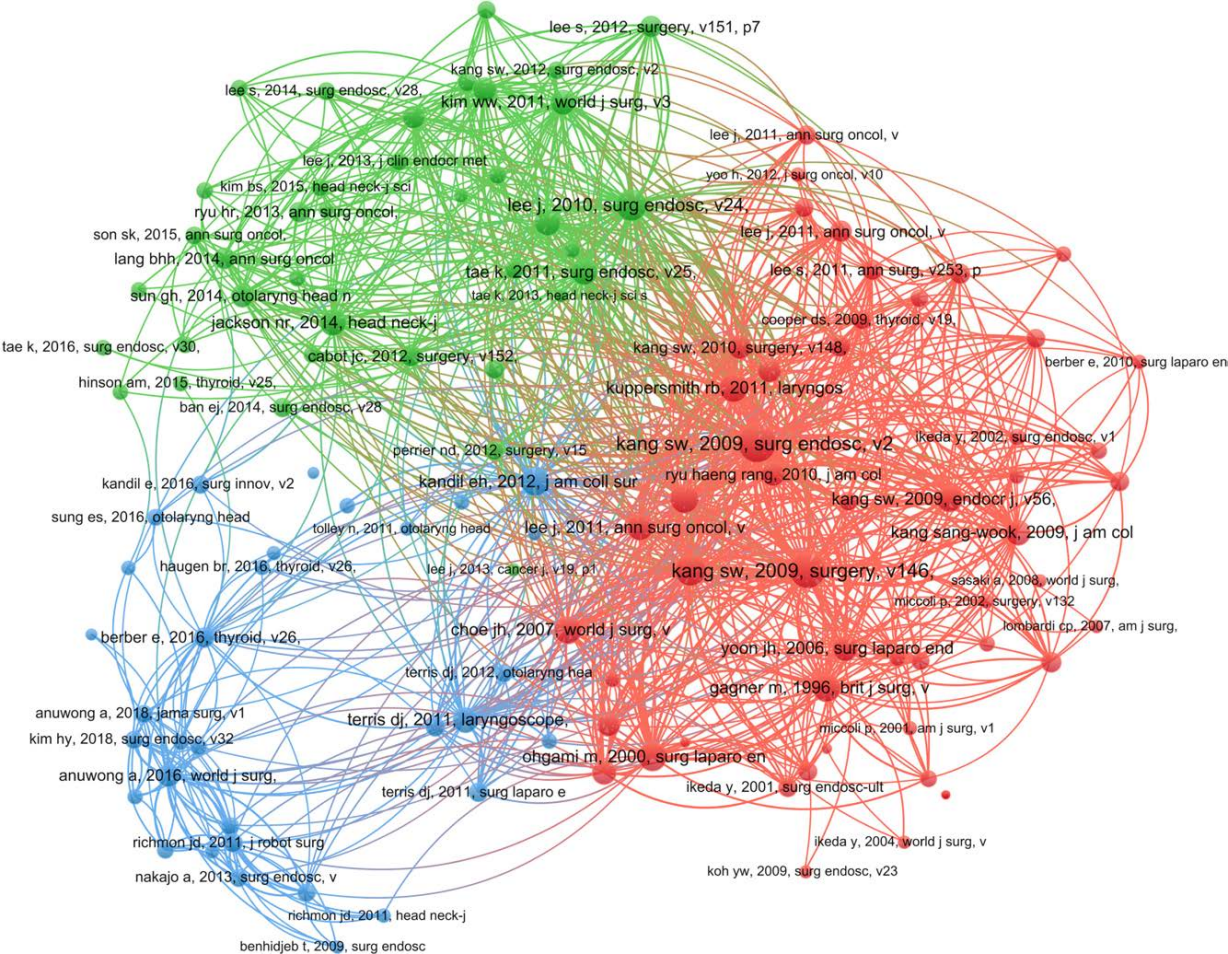
Document co-citation network.

The analysis of the top 10 most frequently cited papers revealed that the paper with the highest GCS score was published by Professor Kang, S. W. from Yonsei University in South Korea in 2009.^[11]^ Additionally, the paper published by Professor Kang, S. W. in 2009 from Yonsei University in South Korea^[23]^ achieved the highest LCS score. Co-cited references serve as an indicator of the degree of association among the references and encompassed 5361 co-cited references within the 505 articles. The most frequently cited paper, authored by Professor Kang, S. W. from Yonsei University in South Korea in 2009,^[23]^ has been co-cited 164 times, indicating its milestone contribution to the development of the field, as detailed in Table 5 and Figure 7.

**Table 5:** Top 10 most cited documents.

Rank	Corresponding authors	Journal Publication	Year	GCS	LCS	GCS perYr	LCS perYr
1	Kang SW	Surgery	2009	300	157	21.43	11.21
2	Kang SW	Surg Endosc	2009	254	164	18.14	11.71
3	Lee J	Surg Endosc	2010	153	108	11.77	8.31
4	Kandil EH	J Am Coll Surgeons	2012	140	84	12.73	7.64
5	Kuppersmith RB	Laryngoscope	2011	132	87	11.00	7.25
6	Anuwong A	Surg Endosc	2018	128	17	25.60	3.40
7	Lee J	Ann Surg Oncol	2011	121	63	10.08	5.25
8	Kang SW	Surgery	2010	119	61	9.15	4.69
9	Terris DJ	Laryngoscope	2011	116	66	9.67	5.50
10	Berber E	Thyroid	2016	115	49	16.43	7.00

GCS = global citation score, LCS = local citation score.

### 3.8. Keyword analysis

**Figure 8.**
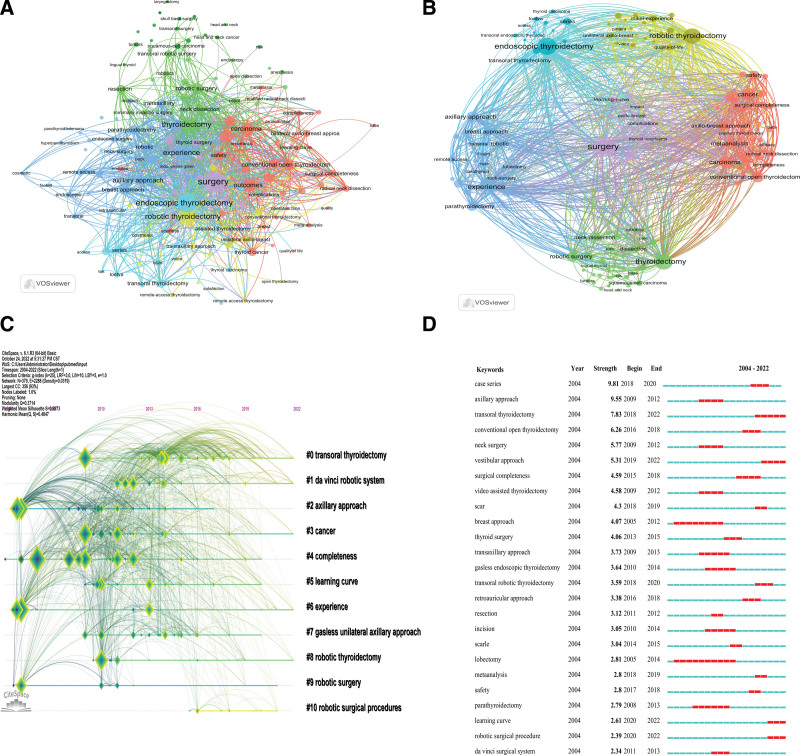
Analysis of keywords related to robot-assisted thyroid surgery. (A) Co-occurrence network of keywords. (B) Clustered co-occurrence network of keywords (divided into 6 categories with different colors). (C) Temporal cluster analysis of keywords. (D) Emphasis analysis of the top 25 keywords with the highest occurrence frequency.

Co-occurrence network analysis was conducted on keywords related to robot-assisted thyroid surgery, extracting 160 keywords by setting a co-occurrence frequency threshold of 5 (Fig. 8A). The top 3 keywords with the highest co-occurrence frequency were “endoscopic thyroidectomy,” “surgical procedures,” and “axillary approach,” indicating the current research hotspots. Cluster analysis identified 6 clusters (Fig. 8B), with the red cluster containing the most keywords (42), covering topics such as conventional open thyroidectomy, surgical integrity, cancer, safety, efficacy, meta-analysis, axillary approach, and neck dissection. The green cluster (33 keywords) mainly focused on thyroidectomy, robotic surgery, neck dissection, resection, and squamous cell carcinoma. The blue cluster (29 keywords) highlighted experience, parathyroidectomy, axillary approach, chest approach, transoral approach, and neck surgery. The yellow cluster (24 keywords) included topics like robot-assisted thyroidectomy, initial experience, unilateral axillary breast, quality of life, and patients. The purple cluster (19 keywords) involved surgery, LC, thyroid tumors, and axillary breast. The light blue cluster (13 keywords) featured endoscopic thyroidectomy, transoral thyroidectomy, transoral endoscopic thyroid surgery, and thyroid cancer. Keywords in the red, yellow, and purple clusters primarily related to clinical research on thyroid surgery, while those in the green, blue, and light blue clusters reflected surgical approaches and entry routes. Temporal cluster analysis using CiteSpace divided the keywords (Fig. 8C) into 11 clusters, representing different research areas and directions. Early research hotspots included the axillary approach (cluster 2), experience (cluster 6), and robotic surgery (cluster 9). Mid-term hotspots included degree (cluster 4), LC (cluster 5), gasless unilateral axillary approach (cluster 7), and robotic surgery systems (cluster 10). Recent hotspots comprised Transoral thyroidectomy (cluster 0), da Vinci robotic surgical system (cluster 1), cancer (cluster 3), and robotic thyroidectomy (cluster 8). Finally, emphasis analysis highlighted keywords with burst strength, indicating increased attention in the past 5 years (Fig. 8D). “Transoral thyroidectomy” (burst strength of 7.83), “vestibular approach” (burst strength of 5.31), “LC” (burst strength of 2.61), and “robotic surgery” (burst strength of 2.39) emerged as frequently mentioned keywords, potentially becoming future research hotspots.

## 4. Discussion

This study employed Microsoft Excel, the R software with the bibliometrix package, Citescape, and VOSviewer software for bibliometric and visualization analysis of relevant literature on robot-assisted thyroid surgery from the WOSCC. The yearly publication output in robot-assisted thyroid surgery has exhibited a consistent upward trajectory, with a simultaneous increase in annual citation counts, particularly from the year 2009 onward. This phenomenon may be linked to clinical research conducted by Professor Kang S. W. from Yonsei University in South Korea.^[11]^ In this study, a large-scale clinical investigation was undertaken for the first time to evaluate the efficacy of robot-assisted thyroid surgery. The results demonstrated that robot-assisted thyroid surgery is both safe and feasible for patients with benign or malignant thyroid conditions, laying the groundwork for the swift expansion of this field. Currently, research on robot-assisted thyroid surgery spans 36 countries and regions, with South Korea, the United States, and China leading in publication quantity. However, notable collaboration occurs primarily among a select few countries, suggesting opportunities for broader and deeper international cooperation. Research institutions exhibit a tendency to collaborate more extensively within their own countries, lacking substantial cross-border institutional collaboration. This may impede the establishment of a comprehensive global collaborative network, posing potential challenges to the long-term stability and development of the field.

In the top 10 list of authors with the highest publication volume, Professor CHUNG WY emerged as the leading contributor, boasting significantly higher publication volume, LCS, and GCS than other authors. Professor CHUNG WY research primarily delves into the clinical application of robot-assisted thyroid surgery for thyroid cancer, affirming its safety and feasibility.^[11,23]^ When compared to traditional open thyroid surgery and conventional endoscopic thyroid surgery, robot-assisted thyroid surgery offered superior 3D imaging and cosmetic outcomes.^[24,25]^ Furthermore, postoperative pain and analgesic dosage show no significant increase,^[26]^ contributing significantly to the increasing favorability of robot-assisted thyroid surgery among surgeons and patients with thyroid diseases. Professor CHUNG WY has also validated the feasibility and safety of robot-assisted thyroid surgery in patients with hyperthyroidism and locally recurrent thyroid cancer,^[27,28]^ highlighting its suitability for various conditions like thyroid cancer, hyperthyroidism, and locally recurrent thyroid cancer. However, it crucial to note that these studies are based on patients from South Korea and may not universally represent all thyroid disease patients worldwide, lacking generalizability. It is recommended to conduct similar clinical studies in diverse countries and regions in the future to enhance the global applicability of robot-assisted thyroid surgery.

In a corpus of 505 articles, 179 (35.44%) were published in 6 core journals. The citation analysis of 505 articles showed the paper with the highest GCS score was published by Professor Kang, S. W. from Yonsei University in South Korea in 2009.^[11]^ This groundbreaking study marked the initial application of robot-assisted thyroidectomy in clinical practice, evaluating the pathological characteristics of patients, surgical outcomes, length of hospital stay, complications, and LC, ultimately confirming its safety and feasibility. This paper played a pivotal role in propelling rapid developments in the field post-2009. Simultaneously, the paper with the highest LCS score is authored by Professor Kang, S. W. from Yonsei University in South Korea and was published in 2009.^[23]^ This paper introduced a robot-assisted endoscopic thyroidectomy technique using a gasless transaxillary approach, overcoming limitations of conventional endoscopic surgery in thyroid cancer operations and demonstrating good applicability. The publication of these 2 studies carries profound significance for the pioneering development of the field. It not only charted new frontiers but also steered an increasing number of clinical practitioners toward a heightened focus and consideration of the domain of robotic thyroid surgery. Consequently, this has spurred a paradigm-shifting evolution in the field, particularly since the year 2010. In a meticulous comparative analysis with traditional open thyroid surgery, Lee, J et al discerned that robotic thyroid surgery excels in terms of cosmetic outcomes, reduced postoperative neck discomfort, and alleviated adverse swallowing symptoms when juxtaposed with conventional approaches. Importantly, no statistically significant differences were observed in postoperative pain and complications. In summary, the integration of robotics in thyroid surgery emerges as a transformative approach, mitigating postoperative neck discomfort and alleviating adverse swallowing symptoms for patients. Furthermore, it ensured excellent incision concealment without introducing an elevated risk of postoperative pain and complications.

Keywords are pivotal in summarizing the content of the articles, and their visualization based on co-occurrence frequency is instrumental in analyzing the research hotspots in robotic thyroid surgery.^[29]^ The research focus in robotic thyroid surgery primarily centers around endoscopic thyroidectomy, surgical techniques, and axillary approach. Over the past 2 decades, surgical technology remarkable progress has transformed thyroid surgery from the initial open thyroidectomy to contemporary endoscopic and robot-assisted thyroidectomy. This evolution not only enhances the safety of thyroid surgery but also increases patient acceptance due to the absence of visible scars on the neck.^[30,31]^ Endoscopic thyroidectomy represents a revolutionary advancement over traditional methods, achieving both aesthetic outcomes and effective removal of thyroid tumors.^[32]^ Nevertheless, some limitations persist, such as the absence of articulating instruments and a 2-dimensional view. The introduction of robotic surgical systems helps overcome these limitations, marking another milestone in the development of thyroid surgery.^[33]^ The approaches for robotic thyroid surgery mainly include axillary,^[34]^ transoral,^[35]^ retroauricular,^[36]^ and bilateral areolar bilateral axillary approaches.^[37]^ Among them, the axillary approach is the most widely used, mainly because the axilla has natural skin creases, providing excellent concealment of the incision. In summary, it becomes evident that aesthetic outcomes play a pivotal role in driving the progress of thyroid surgery. The incorporation of endoscopic and robotic-assisted technologies has significantly propelled the evolution of thyroid surgical procedures.^[38]^ Aesthetic outcomes have now emerged as a relatively novel and important quality criterion.

Cluster analysis of keywords serves as a valuable tool for researchers to gain a comprehensive understanding of the current research landscape in robotic thyroid surgery.^[39]^ According to the clustering results, the current research status of robotic thyroid surgery predominantly revolves around clinical evaluation and surgical approaches and techniques. Common clinical evaluation indicators for thyroid surgery encompass safety, efficacy, quality of life, and LC. De Vries et al^[40]^ conducted a meta-analysis comparing minimally invasive techniques (robotic thyroidectomy, endoscopic thyroidectomy, and minimally invasive video-assisted thyroidectomy) with conventional thyroidectomy, considering factors such as operation time, length of hospital stay, recurrent laryngeal nerve injury, and postoperative complications. Their findings indicate that minimally invasive techniques were non-inferior to traditional thyroid surgery in terms of efficacy and safety. In a meta-analysis by Kandil et al,^[38]^ comparing robotic and non-robotic thyroid surgery, it was observed that robotic thyroid surgery had a longer operation time but no significant difference in postoperative complications. This suggested that these clinical indicators play a crucial role in distinguishing between favorable and unfavorable options in thyroid surgery, thereby fostering the advancement of thyroid surgery. The evolution of surgical approaches for thyroid surgery has shifted from neck incisions to extra-neck approaches, such as axillary, breast, oral, and retroauricular approaches. This shift aims to avoid visible scars, aligning with patients’ cosmetic expectations. This underscores the pivotal role of aesthetics as a driving force behind the development of thyroid surgery.^[41]^

Keyword emergence analysis serves as a valuable tool for reflecting the research frontier within a specific period of time.^[42]^ The analysis has indicated that transoral robotic thyroidectomy (TROT) and LC are the current research frontiers in this field. TROT, an innovative natural orifice endoscopic surgery, is gradually emerging as a viable alternative. Russell et al^[43]^ pioneered the application of TROT in treating thyroid cancer, determining that it resulted in no permanent complications and minimal impact on the quality of life. This procedure, employing the oral vestibular approach, offers several advantages, such as completely avoiding skin incisions, leaving no lasting scars, minimizing or preventing recurrent laryngeal nerve injuries, and proving applicable to individuals with a higher body mass index. Additionally, TROT leverages advanced robotic technology, inheriting numerous benefits associated with robotic surgery, including superior precision and accuracy in surgical procedures, a 3-dimensional high-definition view, no significant learning curve, and conservation of the operator physical stamina. With the rise of minimally invasive techniques, the LC has become a fundamental benchmark, especially in the field of surgical procedures.^[44]^ While many studies employ surgical time as the primary criterion for evaluating the LC, it may not necessarily be the most appropriate indicator of surgical proficiency. A plethora of research assesses the LC of thyroid surgery based on criteria such as surgical time, intraoperative blood loss, length of hospital stay, or the incidence and severity of complications.^[45–47]^ Therefore, future research should consider various confounding factors, carefully select appropriate evaluation criteria, and conduct a rational assessment of the LC in thyroid surgery, ultimately fostering technological innovation in this field. In conclusion, TROT and the learning curve are poised to be future research hotspots and trends.

## 5. Conclusions

This study employed bibliometric techniques to visually analyze the pertinent literature on robot-assisted thyroid surgery within the WOSCC. The findings from the country/region analysis underscored South Korea notable and extensive contributions to research in this domain. Keyword analysis further highlighted the prevailing research hotspots, including endoscopic thyroidectomy, surgical procedures, and axillary approach. Notably, TROT and LC emerged as potential future research trends.

## Author contributions

**Conceptualization:** Ya-Yu Sun.

**Formal analysis:** Xiao-Tong Shi, Jun Shen, Ya-Yu Sun.

**Funding acquisition:** Jun Shen.

**Investigation:** Xiao-Tong Shi, Jun Shen.

**Methodology:** Xiao-Tong Shi, Jun Shen, Ya-Yu Sun.

**Project administration:** Xiao-Tong Shi, Ya-Yu Sun.

**Resources:** Xiao-Tong Shi.

**Software:** Jun Shen.

**Supervision:** Ya-Yu Sun.

**Writing – original draft:** Xiao-Tong Shi, Ya-Yu Sun.

**Writing – review & editing:** Xiao-Tong Shi.
